# Differential Energy Metabolism in Skeletal Muscle Tissues of Yili Horses Based on Targeted Metabolomics and Transcriptomics Analysis

**DOI:** 10.3390/biology14121713

**Published:** 2025-11-30

**Authors:** Xueyan Li, Chen Meng, Yuheng Xue, Zhehong Shen, Wanlu Ren, Yaqi Zeng, Jun Meng

**Affiliations:** 1 College of Animal Science, Xinjiang Agricultural University, Urmuqi 830052, China; lixxuey0421@163.com (X.L.); chenmeng0330@126.com (C.M.); xyh9523@126.com (Y.X.); 18858657690@163.com (Z.S.); 13201295117@163.com (W.R.); xjauzengyaqi@163.com (Y.Z.); 2 Xinjiang Key Laboratory of Horse Breeding and Exercise Physiology, Urumqi 830052, China; 3 Horse Industry Research Institute, Xinjiang Agricultural University, Urumqi 830052, China

**Keywords:** Yili horse, skeletal muscle, energy metabolism

## Abstract

Skeletal muscle is the largest organ system in mammals. To investigate the differences in energy metabolism across various skeletal muscles in Yili horses, this study examined muscle fiber type distributions through immunohistochemical staining of muscles, including the splenius, triceps brachii, longissimus dorsi, and gluteus medius. The splenius and gluteus medius muscles, which exhibited the greatest differences in the proportion of slow-twitch fiber area, were selected for further comparison of differential metabolites and transcriptomic expression profiles between slow-twitch and fast-twitch fibers. A total of 27 energy metabolism-related differential metabolites, including pyruvate and lactate, were identified, along with 432 differentially expressed genes, such as PFKM and ALDOA. Additionally, 164 differentially expressed miRNAs, including miR-499 and miR-24-3p, were detected. We found highlighted differences in LDHA expression between the gluteus medius and splenius muscles, which may influence the conversion of fast and slow muscle fibers by modulating the glycolysis/gluconeogenesis pathway. The miRNA-mRNA targeting relationships established here warrant further validation. These findings provide valuable insights into the molecular mechanisms underlying energy metabolism differences in Yili horses.

## Simple Summary

This study systematically analyzed targeted energy metabolites, mRNA, and miRNA in the skeletal muscles of various regions of Yili horses. A total of 432 mRNAs and 164 miRNAs exhibited differential expression. Notably, the differentially expressed genes are primarily involved in glycolysis, as well as the metabolism of fructose and mannose, highlighting the distinct regulatory mechanisms governing the metabolic characteristics of skeletal muscles in different regions of Yili horses. These findings provide valuable insights into the molecular mechanisms underlying muscle fiber type determination.

## 1. Introduction

The Yili horse, an exceptional breed developed in Xinjiang, China, is known for its outstanding athletic performance, yet its capabilities have not been thoroughly studied. However, current research on equine skeletal muscle has primarily focused on differences between sexes [1] and ages [2], with limited investigation into the variations in energy metabolism across different skeletal muscle sites in Yili horses. Energy metabolism encompasses both material metabolism and energy production, with these processes working in coordination. As an endocrine organ [3], skeletal muscle plays a pivotal role in regulating protein synthesis, energy metabolism, and maintaining homeostasis in glucose and lipid metabolism [4]. Skeletal muscle is composed primarily of fast-twitch and slow-twitch fibers. Fast-twitch fibers are crucial for explosive movements and are highly glycolytic, while slow-twitch fibers are adapted for sustained activities and are rich in mitochondria [5]. The regulatory mechanisms governing fiber type composition and differentiation form the molecular foundation for functional differences in muscle performance. Notably, the proportion of slow muscle fibers in the Mongolian horse’s splenius muscle is significantly higher than in the gluteus medius muscle, fast muscle fibers are crucial for animals to achieve short-term high-intensity explosive power which supports the horse’s head and is consistent with the hypothesis that hind limb muscles generate more propulsive force than forelimb muscles during activity [6].

Advances in transcriptome sequencing technology have greatly facilitated the study of gene regulation in muscle molecular mechanisms, providing insights into how genes influence livestock economic traits. Transcriptomic analysis of muscle tissues can identify key biological pathways that influence cellular energy metabolism and fiber type specification; miRNAs play pivotal roles in myogenesis and skeletal muscle development at various stages, significantly affecting muscle mass, fiber type, and muscle-related diseases.

For instance, whole transcriptome sequencing analysis of oxidative muscle (Biceps femoris, BF) and glycolytic muscle (Obliquus externus abdominis, OEA) in yaks revealed that differentially expressed miRNAs are primarily involved in the PPAR signaling pathway, the tricarboxylic acid cycle (TCA cycle), and myofiber type transformation processes, highlighting regulatory differences between oxidative and glycolytic muscles [7]. Li et al. [8] identified multiple DEGs and DEmiRs in fast and slow muscles of black donkeys; for example, eca-miR-193a-5p and eca-miR-370 may regulate myofiber types by targeting genes such as ACTN3 and PKM, influencing actin binding and glycolytic processes. Sempere et al. [9] first investigated miRNAs functional in mammals (humans, mice) and discovered the expression of three miRNAs (miR-1d, miR-133, miR-206) in skeletal muscle. These miRNAs were later termed myomiRs (myo = muscle) [10]. Most myomiR family members are expressed in both cardiac and skeletal muscle, independent of fiber type, and are primarily enriched in type I muscles (e.g., miR-206, miR-208b, miR-499). To date, no studies have found any miRNAs specifically enriched in type II muscles [11]. A growing body of evidence from livestock studies demonstrates that miRNA expression is fiber-type specific. Comparative analyses between fast-twitch fibers (e.g., Longissimus lumborum, Triceps brachii, and Semimembranosus muscles) and slow-twitch fibers (e.g., Psoas major) reveal distinct miRNA expression profiles. These differentially expressed miRNAs play a central role in determining muscle fiber type by regulating key target genes. For example, studies on skeletal muscle development in Duroc × Landrace × Yorkshire pigs have shown that miR-499-5p promotes the formation of oxidative muscle fibers (types I and IIa) by suppressing Sox6 [12]. Similarly, analysis of muscle fiber composition in the longissimus dorsi (LD) of Rongchang (RC) and Large White (LW) pigs revealed higher miR-152 expression in slow-twitch fiber-rich tissues, where it promotes slow-twitch fiber generation by targeting the UCP3 gene [13]. Additionally, miR-196-5p and other miRNAs are specifically upregulated in slow-twitch fibers, suggesting their potential role in fiber type conversion [14]. miR-133a participates in skeletal muscle formation, mitochondrial function, and myofibrillar type conversion [15,16]. Collectively, these findings confirm a close molecular association between skeletal muscle energy metabolism characteristics and myofibrillar composition.

To explore the regulatory mechanisms behind the different phenotypes of muscles, previous research has identified many DEGs in skeletal muscles with varying fiber types. However, current research on myofibrils and energy metabolism has primarily focused on animals such as pigs, chickens, cattle, and sheep [17,18,19], with limited studies on the role of miRNAs in the skeletal muscle of Yili horses. This study investigates skeletal muscle from different regions of the Yili horse using targeted metabolomics and miRNA functional analysis. The goal is to identify differentially expressed metabolites and miRNAs associated with skeletal muscle energy metabolism and muscle fiber differentiation in Yili horses, thereby enhancing our understanding of the molecular mechanisms underlying energy metabolism and muscle fiber type determination in Yili horse skeletal muscle.

## 2. Materials and Methods

### 2.1. Experimental Animals

This study was conducted at a breeding farm in Zhaosu County, Yili Kazakh Autonomous Prefecture, Xinjiang Uygur Autonomous Region. Eleven Yili stallions, aged 5 to 8 years, were selected as subjects. The horses were fasted for 12 h and deprived of water for 6 h prior to slaughter. Immediately following slaughter, the splenius muscle, triceps brachii, longissimus dorsi, and gluteus medius muscles were collected. The collected tissues were rinsed with physiological saline and divided into two portions: one portion was cut into 1 cm^3^ cubes and fixed in 4% paraformaldehyde (Servicebio, Wuhan, China, G1101) for immunohistochemical staining and slide preparation; the other portion was stored in liquid nitrogen for targeted energy metabolomics and transcriptomic sequencing. Based on the immunohistochemical staining results, the two muscle sites with the most significant differences in slow-twitch fiber area proportion the splenius muscle (JJ) and gluteus medius (TZJ) were selected for targeted energy metabolomics and transcriptomic sequencing.

### 2.2. Muscle Section Preparation

Formaldehyde-fixed tissues from the splenius muscle, triceps brachii, longissimus dorsi, and gluteus medius muscles were dehydrated with xylene and graded ethanol, followed by paraffin embedding. Serial paraffin sections of approximately 5 μm thickness were prepared using a microtome. The sections underwent immunohistochemical staining, which included antigen retrieval, 3% hydrogen peroxide treatment, serum blocking, overnight incubation with primary antibody (rabbit, 1:200 dilution, Servicebio, Wuhan, China) at 4 °C, secondary antibody incubation at room temperature, color development, and hematoxylin staining for nuclei. The sections were then dehydrated, cleared, and mounted. The sections were washed with PBS (Sigma, Shanghai, China), immersed in 3% hydrogen peroxide (Servicebio, Wuhan, China) at 25 °C for 25 min, and then rewashed. After incubation with bovine serum albumin (Sigma, Shanghai, China) for 30 min, the excess liquid was removed. Rapid primary antibody (myosin-1, Servicebio, Wuhan, China GB112130, 1:3,000) was added and incubated overnight at 4 °C in a humid chamber. Following three washes, the sections were incubated with HRP-labeled secondary antibody (Servicebio, Wuhan, China, GB23303, 1:500) at room temperature for 50 min. After another PBS wash, the sections were incubated with CY3-TSA (Servicebio, Wuhan, China) in the dark at 25 °C for 10 min. The sections were then washed with TBST (Servicebio, Wuhan, China) and treated with EDTA antigen retrieval solution by microwaving for 10 min. After cooling, the excess liquid was removed again, and sections were incubated overnight at 4 °C in a humid chamber with the secondary antibody (myosin-7, Servicebio, Wuhan, China GB111857, 1:500). Following PBS washing, the sections were incubated at room temperature for 50 min with HRP-labeled secondary antibody (Servicebio, Wuhan, China, GB25303, 1:400). After removing excess liquid, sections were incubated with DAPI solution (Servicebio, Wuhan, China) at 25 °C in the dark for 10 min, then mounted with neutral binder and stored. The sections were observed using a scanner, with two fields of view selected per section (Pannoramic MIDI, 3DHISTECH, Budapest, Hungary). Image ProPlus 6.0 software was used to calculate and analyze the number and area of fast and slow muscle fibers per unit area.

### 2.3. Targeted Energy Metabolite Assay

#### 2.3.1. Sample Preprocessing

Six Yili horses were randomly selected from a group of eleven, and their splenius and gluteus medius muscles were assigned to the JJ and TZJ groups, respectively. To prepare the samples, 500 μL of pre-chilled 70% methanol-water extract (−20 °C; Merck, Darmstadt, Germany) was added to each of the 12 samples, followed by vortexing for 3 min. The samples were then centrifuged at 4 °C, 12,000 rpm for 10 min. A 300 μL aliquot of the supernatant was transferred to a 1.5 mL centrifuge tube and stored at −20 °C for 30 min before a second centrifugation at 4 °C, 12,000 rpm for 10 min. The supernatant (200 μL) was then processed through a protein precipitation plate for instrument analysis, with the final solution stored at −20 °C.

#### 2.3.2. Sample Detection

The sample extracts were analyzed using an LC-ESI-MS/MS system (Waters ACQUITY H-Class, https://www.waters.com/nextgen/us/en.html (accessed on 13 May 2024); MS, QTRAP^®^ 6500+ System, https://sciex.com/ (accessed on 14 May 2024)). The following analytical conditions were employed: HPLC was performed with an ACQUITY UPLC BEH Amide column (2.1 × 100 mm, 1.7 μm) using a solvent system composed of water with 10 mm ammonium acetate (Sigma-Aldrich, St. Louis, MO, USA) and 0.3% ammonium hydroxide (Sigma-Aldrich, Shanghai, China) (A), and 90% acetonitrile/water (Merck, Darmstadt, Germany) (*v*/*v*) (B). The gradient started at 95% B (0–1.2 min), decreased to 70% B (8 min), further to 50% B (9–11 min), and then ramped back to 95% B (11.1–15 min). The flow rate was set to 0.4 mL/min, and the column temperature was maintained at 40 °C with an injection volume of 2 μL.

Mass spectrometry data were processed using MultiQuant 3.0.3 software. Chromatographic peaks corresponding to target compounds were integrated and calibrated against retention times and peak profiles of reference standards to ensure accurate qualitative and quantitative analysis. Multivariate statistical analyses, including Principal Component Analysis (PCA) and Partial Least Squares Discriminant Analysis (PLS-DA), were performed to identify metabolic differences between groups. Differential Metabolites (DMs) were selected based on VIP > 1.0 and *p*-value < 0.05. Functional analyses, including metabolic pathway analysis, were conducted to interpret the biological significance of these metabolites.

### 2.4. Transcriptome Sequencing Analysis

#### 2.4.1. RNA Extraction and Library Construction

**Figure A1:**
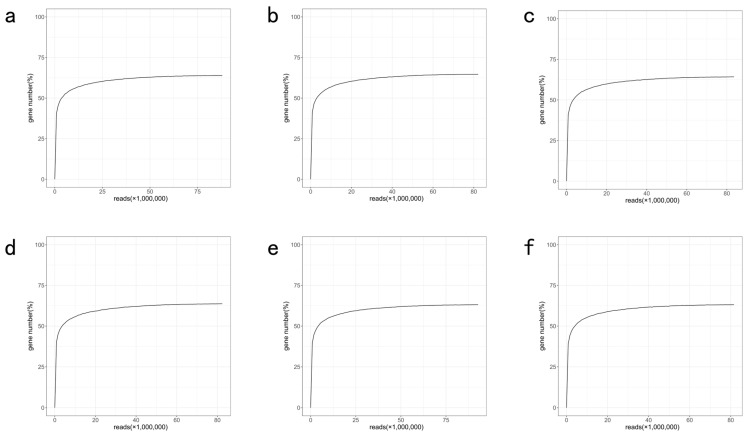
JJ group saturation curve. (**a**–**f**) are the six samples of the JJ group.

**Figure A2:**
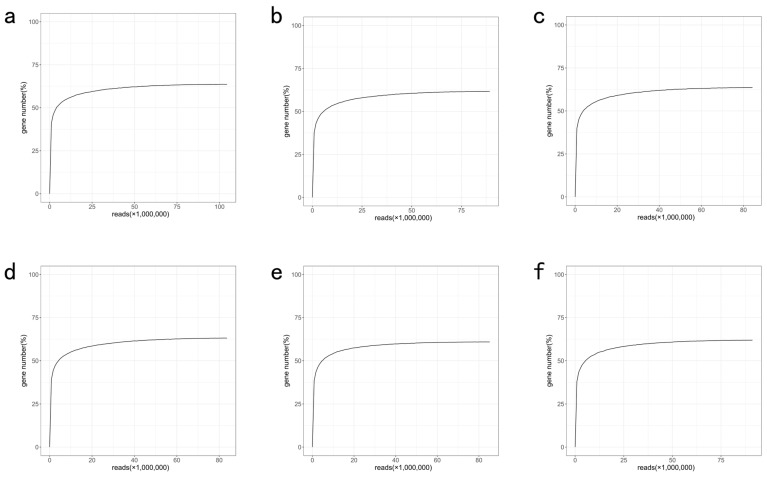
TZJ group saturation curve. (**a**–**f**) are the six samples of the TZJ group.

Total RNA was extracted using Trizol reagent kit (Invitrogen, Carlsbad, CA, USA) according to the manufacturer’s protocol. After total RNA was extracted by Trizol reagent kit (Invitrogen, Carlsbad, CA, USA), the RNA molecules in a size range of 18–30 nt were enriched by polyacrylamide gel electrophoresis (PAGE). Then the 3′ adapters were added and the 36–44 nt RNAs were enriched. The 5′ adapters were then ligated to the RNAs as well. The ligation products were reverse transcribed by PCR amplification and the 140–160 bp size PCR products were enriched to generate a cDNA library and sequenced using Illumina Novaseq6000 by Gene Denovo Biotechnology Co. (Guangzhou, China). The RNA sequencing data quality assessment is provided in Table A1 and Table A2 in Appendix A, the sample saturation curve is shown in Figure A1 and Figure A2 in Appendix A.

#### 2.4.2. mRNA Data Processing and Analysis

Initially, raw sequencing data from each sample underwent processing, including filtering to remove reads containing Ns, low-quality reads, and adapter sequences. After filtering according to the these criteria, valid data (clean reads) were obtained. Subsequent analyses were conducted on these clean reads. Hisat2 (version 2.1.0) software was used to align and annotate the sequencing data from each sample against the equine genome available in the NCBI Genome Database (https://www.ncbi.nlm.nih.gov/datasets/genome/GCF_002863925.1/, (accessed on 5 May 2025). Differential expression analysis was performed using DESeq (version 3.18), with hypothesis testing probabilities (*p*-values) calculated based on the model. Multiple hypothesis testing correction was applied to obtain q-values. DEGs were selected based on q-values and Fold Change, with the criteria for differential expression set to |log_2_(Fold Change)| > 2 and q-value < 0.05. The identified DEGs were annotated by comparison with the GO and KEGG databases to obtain enrichment annotation information.

#### 2.4.3. miRNA Data Processing and Analysis

miRNA data were aligned with sequencing reads using Bowtie (version 2.2.8) software and the Equus asinus reference genome (https://www.ncbi.nlm.nih.gov/genome/?term=Equus+caballus, accessed 24 March 2025). Newly discovered and known miRNAs were identified using the open-source software miRDeep2 (version 0.0.5). Known miRNAs were defined as those matching sequences in the miRBase database for Equus caballus. miRNA expression levels were normalized based on transcripts per million (TPM) after calculation. DEmiRNAs were screened using the DESeq2 tool, with fold change >1.5 and *p*-value < 0.05 as the selection criteria. Target gene prediction was performed using Miranda (v3.3a) and TargetScan (version 7.0). For Miranda, the score threshold was set to 140, the energy threshold to −10 kcal/mol, strict 5′ seed pairing was required, and the gap-open and gap-extend penalties were set to −4.0 and −9.0, respectively. For TargetScan, the 2–8 nt of the 5′ end of the small RNA were used as seed sequences to predict targets in the 3′-UTR region of the transcript. The intersection of the target genes predicted by both methods was used as the final result.

#### 2.4.4. DEGs and DEmiR Enrichment Analysis

All target genes of DEGs and DEmiRNAs were mapped to terms in the Gene Ontology (GO) database (http://www.geneontology.org, accessed 24 March 2025). Use the R package (version 16.2) clusterProfiler to perform functional enrichment analysis on high confidence target genes with MFE ≤ −24 kcal/mol, using all detectable target genes as the background set. Use *p* < 0.05 as the significance threshold to screen for significantly enriched GO terms and KEGG pathways. This analysis enabled the identification of primary biochemical metabolic and signal transduction pathways in which miRNA target genes are involved.

#### 2.4.5. Construction of Metabolite-mRNA-miRNA Interaction Network

A stepwise strategy was applied to construct the metabolite-mRNA-miRNA regulatory network.

(1) Screening of miRNA-mRNA interaction pairs: High-confidence miRNA-mRNA interactions were selected based on the predicted pairing results, using an MFE threshold of ≤−24 kcal/mol.

(2) mRNA-metabolite correlation analysis: Pearson correlation analysis was performed in RStudio (version 1.2.5033) to evaluate associations between mRNAs and metabolites. Significant correlations were identified using thresholds of |r| > 0.65 and *p* < 0.05.

(3) Network integration and visualization: High-confidence miRNA-mRNA and mRNA–metabolite relationships were integrated and imported into Cytoscape (version 3.9.1) to construct and visualize the metabolite-mRNA-miRNA interaction network.

#### 2.4.6. Real-Time Fluorescent Quantitative PCR

Seven DEGs were randomly selected from the transcriptome sequencing results for qRT-PCR analysis with 18S rRNA used as the internal reference gene. Total RNA was reverse-transcribed to cDNA using the PrimeScript RT Reagent Kit (TaKaRa, Japan, Cat. No. 6210). Primers were designed using Primer Premier 5.0 (Table 1). PCR reactions were performed in a 15 μL system containing 7.5 μL 2× qPCR Mix, 1.5 μL of forward and reverse primers, 2.0 μL cDNA, and 4.0 μL nuclease-free water. The amplification protocol included an initial denaturation at 95 °C for 30 s, followed by 40 cycles of 95 °C for 15 s and 60 °C for 30 s, with fluorescence signal collected at 0.5 °C increments. Relative gene expression levels were calculated using the 2^−ΔΔCt^ method and log_2_-transformed prior to analysis. The Real-Time Fluorescent Quantitative PCR experimental steps is provided in Table 2. 

### 2.5. Statistical Analysis

Experimental data were analyzed statistically using Excel 2010, with plots generated in GraphPad Prism (v8.0.1.244) One-way ANOVA was performed using SPSS 24.0 (IBM, Armonk, NY, USA) to assess differences in skeletal muscle fibers across various regions of Yili horses. Results are expressed as “mean ± standard error of the mean (Mean ± SEM).” Lowercase letters indicate significant differences, while uppercase letters denote highly significant differences.

## 3. Results

### 3.1. Differences in Skeletal Muscle Fibers Among Different Regions of the Yili Horse

**Figure 1:**
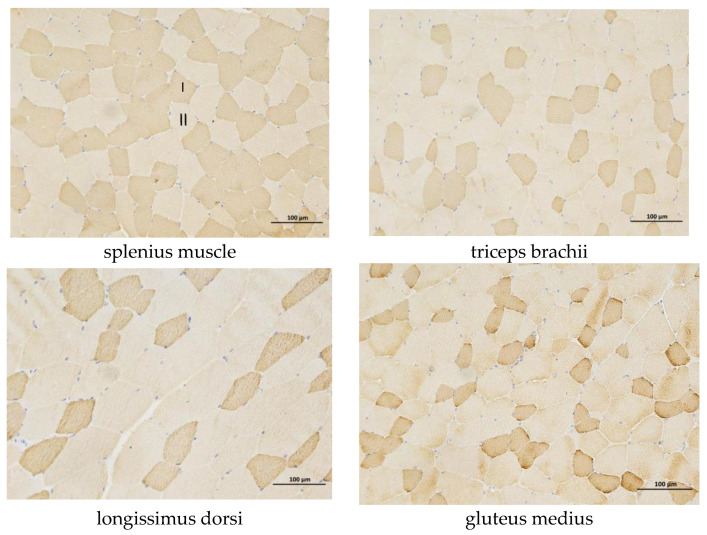
Skeletal muscle sections from different regions of the Yili horse. In the figure, I represents slow muscle fibers and II represents fast muscle fibers, bar = 100 μm.

As shown in Table 3, under identical field-of-view conditions, the proportion of slow-twitch muscle fibers in the triceps brachii, longissimus dorsi, and gluteus medius was significantly higher than that in the splenius muscle (*p* < 0.01). The mean area of slow-twitch fibers in the gluteus medius was significantly smaller than in the adductor magnus (*p* < 0.01). Based on these findings, subsequent studies focused on the TZJ (fast-twitch muscle) and JJ (slow-twitch muscle). The skeletal muscle sections from different regions of the Yili horse show in Figure 1.

### 3.2. Targeted Energy Differential Metabolite Determination

#### 3.2.1. Grouped Principal Component Analysis and OPLS-DA

**Figure 2:**
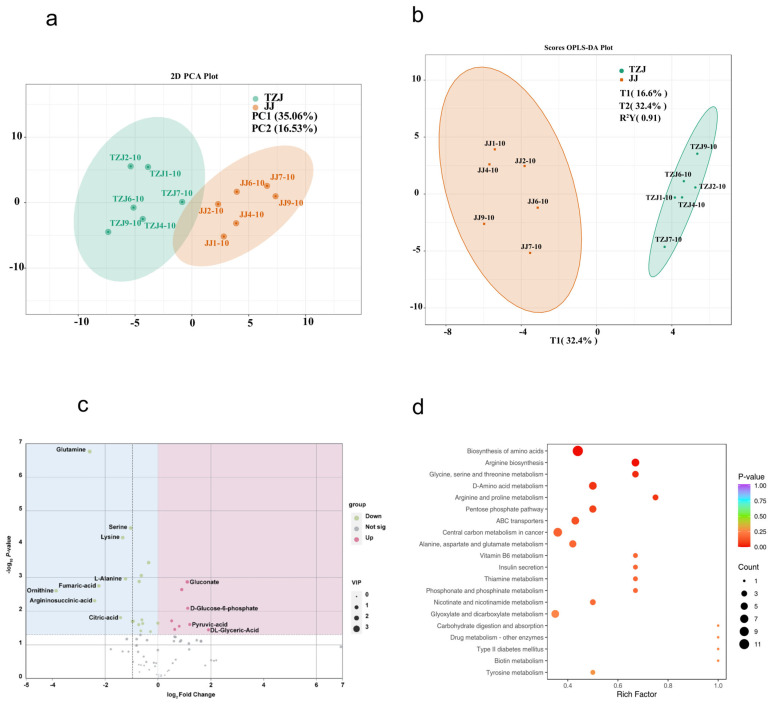
(**a**) PCA plot, where PC1 represents the first principal component, PC2 represents the second principal component, and the percentages indicate the explanatory power of these principal components for the dataset. Each point represents a sample, with samples from the same group depicted in the same color. Grouping is indicated in the legend. (**b**) S-plot, where the horizontal axis represents the covariance between principal components and metabolites, and the vertical axis represents the correlation coefficient between principal components and metabolites. Metabolites closer to the upper right and lower left corners show more significant differences, with red dots indicating VIP values greater than 1 and green dots indicating VIP values less than or equal to 1. (**c**) Volcano plot of DMs, where green dots indicate down-regulated metabolites and red dots indicate up-regulated metabolites. (**d**) KEGG pathway bubble plot of DMs, with KEGG pathway names on the vertical axis and enrichment factor on the horizontal axis. The size of the dots represents the number of enriched metabolites, and the color of the dots indicates different ranges of *p*-values.

Principal component analysis of metabolites revealed a clear separation between the JJ and TZJ groups (Figure 2a), supporting the appropriateness of the control group design for subsequent metabolomic analyses. In addition, permutation testing based on orthogonal partial least squares discriminant analysis (OPLS-DA) confirmed the model’s strong predictive performance and discriminative power (JJ vs. TZJ: R^2^Y = 0.91), effectively excluding the possibility of overfitting and demonstrating substantial metabolic divergence between the two phenotypes (Figure 2b).

#### 3.2.2. Differential Metabolite Analysis

This study identified 27 DMs between JJ and TZJ, including 9 up-regulated and 18 down-regulated metabolites. These metabolites include ornithine, glycerate, L-cysteine, serine, L-glutamate, gluconate, pyruvate, lactate, D-ribose-5-phosphate, D-mannose-6-phosphate, and D-glucose-6-phosphate (Table 4).

#### 3.2.3. KEGG Analysis of Differential Metabolites

As shown in Figure 2d, the DMs between JJ and TZJ are involved in 80 metabolic pathways. These pathways were significantly enriched in KEGG categories such as the pentose phosphate pathway, pyruvate metabolism, and glycolysis or gluconeogenesis.

### 3.3. Transcriptome Sequencing Analysis Result

#### 3.3.1. mRNA and miRNA Differential Expression Analysis

**Figure 3:**
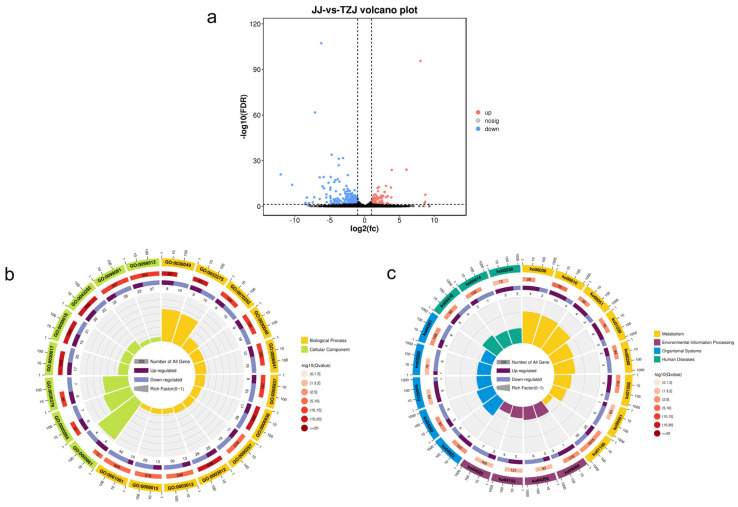
(**a**) mRNA volcano plot, where blue indicates down-regulation and red indicates up-regulation; (**b**) mRNA GO enrichment circle diagram: First circle: Top 20 enriched GO terms. The scale outside the circle represents the number of differentially expressed genes, with different colors representing distinct Ontologies. Second circle: The number of differentially expressed genes relative to the background count for each GO term, along with the Q-value. Longer bars indicate a higher background count, while redder colors correspond to lower Q-values. Third circle: Bar chart showing the proportion of up- and down-regulated differentially expressed genes. Dark purple represents up-regulated genes, while light purple represents down-regulated genes; specific values are shown below. Fourth circle: RichFactor values for each GO term (calculated as the number of differentially expressed genes in that GO term divided by the total number of genes in that term), with background grid lines where each grid represents 0.1. (**c**) KEGG enrichment circle diagram for mRNA: First circle: Top 20 enriched pathways, with the scale outside the circle indicating the number of differentially expressed genes. Different colors represent different pathway classes. Second circle: The number of differentially expressed genes in the background for each pathway and its Q-value. Longer bars indicate a larger number of differentially expressed genes in the background, and redder colors indicate smaller Q-values. Third circle: Bar chart showing the proportion of up- and down-regulated differentially expressed genes. Dark purple represents up-regulated genes, while light purple represents down-regulated genes; specific values are displayed below. Fourth circle: RichFactor values for each pathway (calculated as the number of differentially expressed genes in the pathway divided by the total number of genes in the pathway), with background grid lines where each grid represents 0.1.

**Figure 4:**
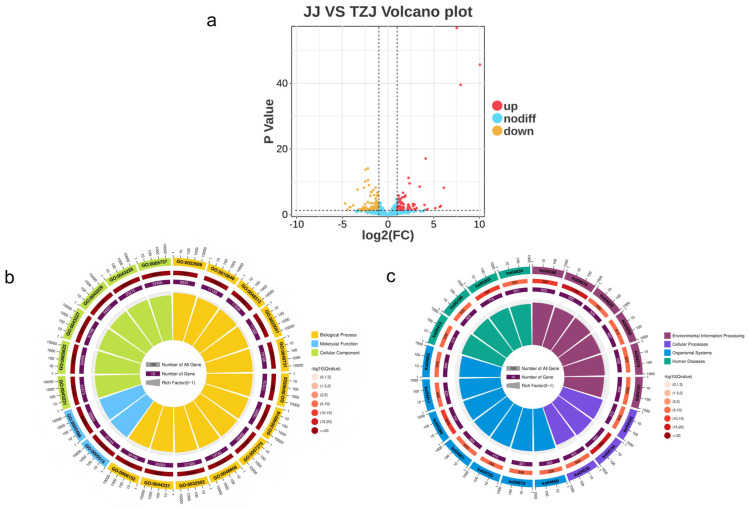
(**a**) miRNA target gene volcano plot, where yellow indicates down-regulation and red indicates up-regulation. (**b**) GO enrichment circle diagram for miRNA target genes: First circle: Top 20 enriched GO terms, with the scale outside the circle indicating the number of miRNA target genes. Different colors represent distinct Ontologies. Second circle: The number of miRNA target genes in the background for each GO term and its Q-value. Longer bars indicate more background genes, while redder colors represent smaller Q-values. Third circle: The number of miRNA target genes for each GO term. Fourth circle: RichFactor values for each GO term (calculated as the number of miRNA target genes in that GO term divided by the total number of genes in that term), with background grid lines, where each grid represents 0.1. (**c**) KEGG enrichment circle diagram for miRNA target genes: First circle: Top 20 enriched pathways, with the scale outside the circle indicating the number of miRNA target genes. Different colors represent different pathway classes. Second circle: The number of miRNA target genes in the background for each pathway and its Q-value. Longer bars indicate a greater number of miRNA target genes in the background, and redder colors represent smaller Q-values. Third circle: The number of miRNA target genes in each pathway. Fourth circle: RichFactor values for each pathway (calculated as the number of miRNA target genes in the pathway divided by the total number of genes in the pathway), with background grid lines where each grid represents 0.1.

Further analysis of mRNAs using the criteria |log_2_(Fold Change)| > 2 and q < 0.05 identified 432 DEGs between JJ and TZJ, including 100 up-regulated and 332 down-regulated genes (Figure 3a). Similarly, applying |log_2_(Fold Change)| > 2 and *p* < 0.05 as screening criteria, a total of 164 DEmiRNAs were identified comprising 73 up-regulated and 91 down-regulated miRNAs (Figure 4a).

#### 3.3.2. Enrichment Analysis of Target Genes for Differentially Expressed mRNAs and miRNAs

GO database annotation of DEGs in JJ vs. TZJ revealed 1761 significantly enriched GO terms (Q < 0.05) among 7611 terms, with significant enrichment in muscle contraction, striated muscle contraction, muscle structure development, contractile fibers, and myofibrils (Figure 3a). Functional annotation of miRNA target genes in JJ vs. TZJ identified 483 significantly enriched GO terms (Q < 0.05) from 7146 terms, including muscle contraction, muscle system processes, striated muscle contraction, muscle filament sliding, and striated muscle tissue development (Figure 4b).

KEGG pathway analysis was conducted on the target genes of differentially expressed mRNAs and miRNAs between JJ and TZJ to identify key pathways involved in energy metabolism across different muscle tissues. DEGs were significantly enriched in pathways related to glycolysis/gluconeogenesis, glucagon signaling, the pentose phosphate pathway, fructose and mannose metabolism, HIF-1 signaling, AMPK signaling, PPAR signaling, and cGMP-PKG signaling (Q < 0.05) (Figure 3c and Figure 4c).

Target genes of DEmiRNAs in JJ vs. TZJ were also significantly enriched in glycolysis/gluconeogenesis, glucagon signaling, amino acid biosynthesis, fructose and mannose metabolism, AMPK signaling, and PPAR signaling pathways (Q < 0.05). Notably, LDHA was involved in three pathways, PFKL in eight pathways, and PKM in five pathways.

#### 3.3.3. mRNA Protein–Protein Interactions (PPIs)

**Figure 5:**
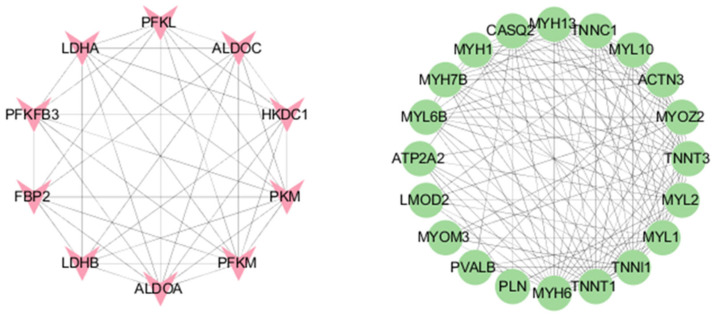
Protein-protein interaction network of hub genes. The network was constructed using the top 30 hub genes identified from the RNA-seq dataset. Hub genes were prioritized with the CytoHubba plugin in Cytoscape, and the top 10 genes ranked by the MCC algorithm-which are all associated with energy metabolism-are shown in the left panel. The remaining 20 genes from the top 30 are presented in the right panel. Protein-protein interaction data were obtained from the STRING database, and only interactions with a confidence score > 0.45 were retained as reliable connections.

Protein interactions in the STRING database are represented by confidence values, with interactions with a confidence score greater than 0.45 considered valid network connections. Hub genes were identified using the CytoHubba plugin in Cytoscape (version 3.9.1), selecting the top 10 genes based on maximum clique centrality (MCC) scores, which included PFKL, ALDOC, HKDC1, PKM, PFKM, ALDOA, LDHB, FBP2, PFKFB3, and LDHA (Figure 5).

#### 3.3.4. Correlation Analysis of DEGs and DMs

**Figure 6:**
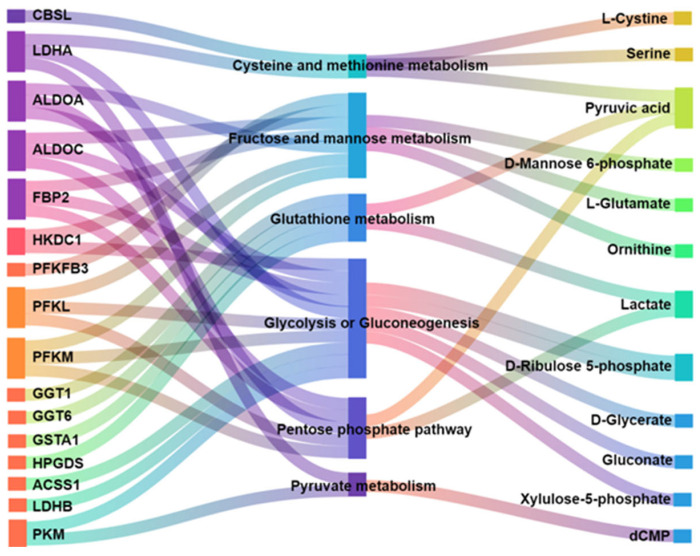
Correlation analysis between DEGs and DMs.

To elucidate the synergistic regulatory mechanisms underlying energy metabolism in the skeletal muscle of Yili horses, we performed an integrated analysis combining transcriptomic data with targeted energy metabolomics. KEGG pathway enrichment analysis was first conducted to identify pathways significantly enriched for both DEGs and DEMs (*p* < 0.05). The corresponding DEGs and DEMs from these pathways were subsequently extracted and visualized using a Sankey diagram. In total, 16 DEGs and 12 DEMs were jointly enriched in six key energy metabolism pathways: cysteine and methionine metabolism, fructose and mannose metabolism, glutathione metabolism, glycolysis/gluconeogenesis, the pentose phosphate pathway, and pyruvate metabolism (Figure 6).

#### 3.3.5. Construction of miRNA-mRMA-Metabolite Network

**Figure 7:**
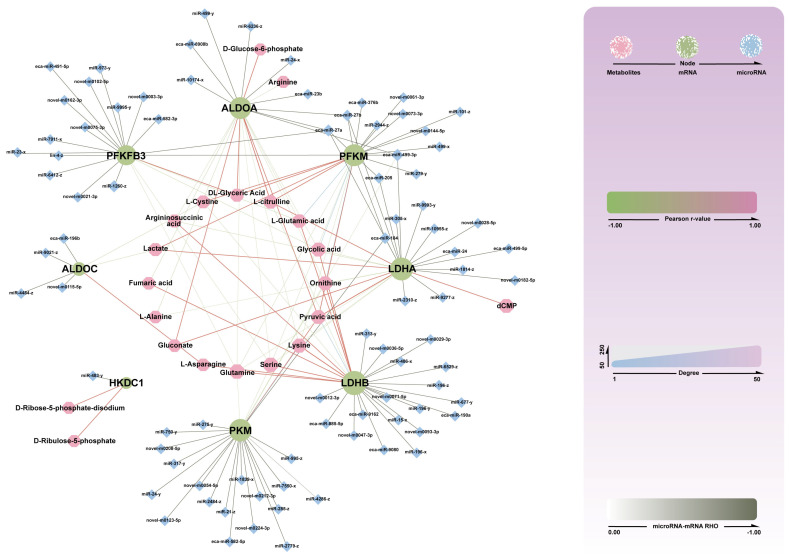
mRNA-miRNA-metabolite interaction network diagram. Pink circles represent metabolites, green circles represent mRNAs, and blue diamonds represent miRNAs. Thicker lines between mRNAs and miRNAs indicate stronger correlations; red lines between mRNAs and metabolites represent significant positive correlations, while green lines indicate significant negative correlations, with line thickness indicating the strength of the correlation.

In this study, 8 key target genes were predicted based on 350 DMs, all of which represent differentially expressed mRNAs negatively correlated with miRNA targets. Among these, miRNAs such as miR-24, miR-27a, miR-27b, miR-499-3p, miR-499-5p, and miR-205 exhibited high connectivity. Specifically, miR-24 and miR-499-5p regulate LDHA; miR-27a and miR-27b regulate ALDOA, PFKM, and LDHA; miR-499-3p regulates ALDOA, PFKM, PFKFB3, and LDHA; and miR-205 regulates PFKM and LDHA. In terms of metabolites, ALDOA correlates positively with glycerate, gluconate, citrulline, and pyruvate; PFKFB3 correlates positively with glycerate; PFKM is positively correlated with glycerate, L-cysteine, lactate, citrulline, and pyruvate; while LDHA shows positive correlations with citrulline, lactate, gluconate, pyruvate, and dCMP. These miRNA-mRNA-metabolite targeting relationships are likely to play a significant role in energy metabolism processes across different skeletal muscles in Yili horses, meriting further investigation (Figure 7).

#### 3.3.6. RT-qPCR Validation of mRNAs

**Figure 8:**
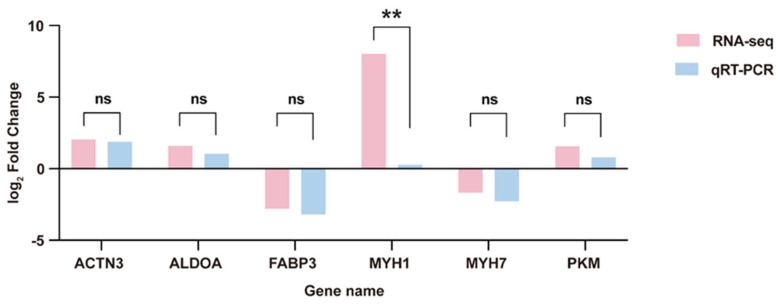
qRT-PCR results of differentially expressed genes. ns represents no significant difference and “**” represents extremely significant difference.

The RNA samples used for qRT-PCR were the same as those from the transcriptome sequencing. Six DEGs were randomly selected from the transcriptome sequencing results. Validation confirmed that the qRT-PCR trends aligned with the RNA-seq findings (Figure 8). This demonstrates the reliability of the RNA-seq data analysis in this study, supporting its use in subsequent research.

## 4. Discussion

Metabolites play a fundamental role in phenotype formation, and analyzing their composition is critical for understanding the intrinsic mechanisms underlying specific biological traits. miRNAs regulate various biological processes in muscle, including myofiber morphology and structural development. Integrating transcriptomic and targeted metabolomic analyses to explore energy metabolism differences across skeletal muscle sites in Yili horses provides deeper insights into the molecular networks that govern muscle function and myofiber composition, offering critical data to understand skeletal muscle fiber diversity in Yili horses.

The pathways involved in the DEGs, DEmiRNAs, and DMs identified in this study include the pentose phosphate pathway, pyruvate metabolism, glycolysis/gluconeogenesis, fructose and mannose metabolism, AMPK signaling, and PPAR signaling pathways. The primary pathways involved in ATP production within skeletal muscle include the ATP phosphagen system, anaerobic glycolysis, and aerobic metabolism (which consists of glycolysis and fatty acid β-oxidation) [20]. Glycolysis/gluconeogenesis is an essential pathway for energy production in skeletal muscle, supporting basic functions [21]. Fructose and mannose metabolism supply substrates for nucleoside synthesis and are closely linked to glycolysis. The interconversion of fructose-6-phosphate and mannose-6-phosphate is crucial for maintaining the substrate balance necessary for glycolysis and glycosylation reactions [22]. In the present study, DEGs primarily clustered within these two pathways, likely because Type I fibers constitute a larger proportion of the muscle fiber area in JJ compared to TZJ. Type I fibers have lower glycogen content and exhibit reduced activity of glycolytic enzymes, such as lactate dehydrogenase (LDHA) and pyruvate kinase [23]. Consequently, pyruvate and lactate levels are higher in the TZJ group. Muscle glycogen is converted to glucose-6-phosphate via glycogen phosphorylase (PHOS), or glucose (GLU) from the blood enters the glycolytic pathway through phosphorylation by hexokinase (HK). The final product, pyruvate (PYR), is then reduced to lactate (LAC) by LDH, generating ATP through substrate-level phosphorylation.

Based on PPI, we selected the top ten genes with MCC scores, which are all related to energy metabolism pathways, facilitating our subsequent research. By constructing a metabolite-mRNA interaction network, ALDOA, PFKM, LDHA, and PFKFB3 showed strong connectivity and positive correlations with pyruvate, glycerate, and lactate. These genes-ALDOA, PFKM, LDHA, and PFKFB3-were identified as key regulators of differential energy metabolism in the skeletal muscles of Yili horses. Fructose-1,6-bisphosphate aldolase (ALDOA), a glycolytic enzyme, catalyzes the reversible conversion of fructose-1,6-bisphosphate to 3-phosphoglycerate, and is encoded by the ALDOA gene [24]. Phosphofructokinase (PFK) is a key regulatory enzyme in glycolysis and serves as the pacemaker of the glycolytic process. In mammals, PFK comprises three isoforms: PFKM, PFKL, and PFKP, each exhibiting distinct regulatory mechanisms and catalytic properties. PFKM regulates glucose metabolism and maintains various biological processes, including muscle function. It is a critical rate-limiting enzyme in glycolysis and glucose metabolism [21]. PFKM catalyzes the irreversible conversion of fructose-6-phosphate to fructose-1,6-bisphosphate, playing a regulatory role in growth, development, and organogenesis [25]. LDHA is essential for lactate metabolism, regulating the interconversion between lactate and pyruvate, and maintaining lactate concentrations [26]. Jeong-An Gim et al. [27] found that LDHA and GSY1 were downregulated in purebred horses after exercise to ensure efficient post-exercise lactate metabolism, preventing lactate accumulation and facilitating energy conversion. PFKFB3, as the rate-limiting enzyme of glycolysis, serves as the primary enzyme sensing glucose concentration in skeletal muscle cells. Its activity significantly regulates systemic metabolic functions. By generating F-2,6-bisphosphate glucose (F-2,6-BP), it drives cellular differentiation toward the glycolytic pathway [28]. AMPK promotes glucose uptake and glycolysis by activating phosphofructokinase (PFKFB) [29], while simultaneously inhibiting glycogen synthesis [30]. ALDOA, PFKM, LDHA, and PFKFB3, as key genes directly involved in the glycolytic pathway, exhibited higher expression levels in the TZJ group than in the JJ group in this study. This is likely because TZJ, as a fast-twitch muscle, primarily relies on anaerobic glycolysis for energy supply [31].

Multiple studies indicate that skeletal muscle development is regulated by complex multimodal, multilevel, and multidimensional molecular targets and genetic mechanisms [32]. As key regulators, miRNAs play vital roles in various biological processes. miRNAs, a class of non-coding small RNAs (~22 nucleotides in length), are found in eukaryotes and typically bind to the 3′ UTR region of target mRNAs, leading to mRNA degradation or inhibition of post-transcriptional translation processes [33]. The specific role of miRNAs in skeletal muscle development in Yili horses remains unclear. However, studies suggest that certain key miRNAs influence muscle development, particularly during growth, significantly affecting muscle fiber size and type [34].

In this study, miRNAs such as miR-24, miR-27a, miR-27b, miR-499-3p, miR-499-5p, and miR-205 exhibited high connectivity. Notably, LDHA was identified as a common target for miR-24, miR-499-3p, miR-499-5p, and miR-205. ZHANG et al. [35] suggested that LDHA enhances myocyte differentiation by promoting glycolysis and the TCA cycle. Mechanistically, LDHA facilitates glycolysis and the TCA cycle by enhancing the NADH cycle while inhibiting oxidative phosphorylation in the electron transport chain (ETC), thereby supplying intermediate metabolites that support myocyte differentiation. Additionally, increased ATP production from glycolysis via LDHA induces Akt phosphorylation, activating the PI3K-Akt signaling pathway, which further aids myocyte differentiation. Jideng Ma et al. [32] investigated the role of the myomaker gene in porcine skeletal muscle development, cellular differentiation, and muscle injury repair. They found that miR-205 overexpression significantly suppressed Myomaker expression and fusion function, inhibiting MyHC-2x expression in different myofibrillar types. This suggests that miR-205 also influences myofibrillar differentiation. Qiang Sun et al. [36] observed that miR-24 expression was significantly upregulated during myoblast differentiation and could be inhibited by TGF-β1. Their experiments confirmed that TGF-β1 suppresses miR-24 transcription via Smad3-dependent mechanisms and binding sites in the miR-24 promoter region. In Smad3-deficient myoblasts, TGF-β1 failed to suppress miR-24 expression, accelerating myogenesis. Thus, miR-24 is considered a TGF-β1-regulated miRNA involved in skeletal muscle differentiation. miR-24-3p, a major mature product of the miR-24 gene cluster, plays a pivotal role in myogenesis and myofibrillar type conversion. Danyang Fan et al. [37] demonstrated that miR-24-3p suppresses muscle cell proliferation while promoting differentiation, facilitates the transformation of slow-twitch fibers into fast-twitch fibers, and influences the expression of the glucose transporter GLUT4. Pengfei Wu et al. [38] identified downstream target genes of miR-24-3p (Notch1, CTNNB1, and RYR3) that are closely associated with muscle growth and development. In our study, we found that miR-499-3p and miR499-5p were highly expressed in splenius muscle (enriched in slow-twitch fibers) compared to gluteus medius (enriched in fast-twitch fibers). This suggests that these miRNAs may be involved in the regulation of slow-twitch fiber properties. Our findings are consistent with previous reports in other species. For example, the upregulation of miR-499-3p and miR499-5p in slow-twitch muscles has also been observed in mouse Soleus muscle [11]. Van Rooij et al. [39] demonstrated that miRNAs regulate skeletal muscle fiber type by suppressing Sox6 expression. miR-208b and miR-499 promote slow-twitch fiber formation by inhibiting Sox6, and simultaneous inactivation of both miR-208b and miR-499 significantly reduces type I fiber abundance in the soleus muscle. Gan et al. [40] found that ERRγ stimulates the expression of miR-499 and miR-208b, which increases the proportion of type I fibers. The miR-27 family consists of miR-27a and miR-27b, which are located on distinct chromosomes (miR-27a on human chromosome 19 and mouse chromosome 8, miR-27b on human chromosome 9 and mouse chromosome 13) [41]. Crist et al. [42] found that miR-27b promotes the initiation of differentiation in both satellite cells and regenerative skeletal muscle by downregulating Pax3 protein expression. Huang et al. [43] proposed that miR-27a participates in myoblast differentiation during skeletal muscle formation. In this study, the relationship between miR-27a and miR-27b and their respective target genes requires further confirmation.

Consistent with observations in rodents [39] and Mongolian horse [44], we found that miR-499-3p and miR-499-5p were abundantly expressed in Yili horse splenius muscle, underscoring their evolutionarily conserved role in fundamental processes such as myogenesis and muscle maintenance. To our knowledge, this study represents the first comprehensive systematic investigation of miRNA expression profiles across skeletal muscle tissues from different tissues of the Yili horse. As a native Chinese horse breed renowned for its exceptional environmental adaptability, ability to thrive on coarse feed, and outstanding athletic performance, the Yili horse holds significant research value for in-depth analysis of its molecular regulatory mechanisms, particularly the role of miRNAs. Our data establish a robust foundation for subsequent research, enabling the association of these unique miRNA expression patterns with the Yili horse’s valuable phenotypic traits, such as athletic performance and meat quality. This original dataset provides a valuable resource for equine genetics research and may offer scientific support for selective breeding programs aimed at enhancing specific muscle-related traits in horses. This study has certain limitations, such as insufficient sample size and gender homogeneity. We will expand the sample size and conduct further research on horses of different genders and age groups to explore the universal applicability of these molecular markers.

## 5. Conclusions

This study identified 27 differentially targeted energy metabolites, 432 DEGs, and 164 DEMs in JJ and TZJ. Key pathways involved include the pentose phosphate pathway, pyruvate metabolism, glycolysis or gluconeogenesis, and fructose and mannose metabolism. By constructing a metabolite-mRNA-miRNA interaction network, mRNAs (e.g., ALDOA, PFKM, PFKFB3, LDHA) and miRNAs (e.g., miR-23b, miR-24, miR-27a, miR-27b, miR-499-3p, miR-499-5p, and miR-205) were identified as potential regulators of energy metabolism and myocyte differentiation in Yili horse skeletal muscle. We found that highlighted differences in LDHA expression between the gluteus medius and splenius muscles, which may influence the conversion of fast and slow muscle fibers by modulating the glycolysis/gluconeogenesis pathway. The miRNA-mRNA targeting relationships established here warrant further validation. These findings provide valuable insights into the molecular mechanisms underlying energy metabolism differences in Yili horses.

## Figures and Tables

**Table 1:** Primers used in real-time fluorescence quantitative PCR.

Gene ID	Gene	Primer Name	Primer Sequence	Product Size (bp)	Accession No.
100033871	*ACTN3*	ACTN3-F	GGTGAACCAGGAAAACGA	160	NM_001163869.1
		ACTN3-R	CCGGTAGTCCCGAAAGTC	160	
100033895	*FABP3*	FABP3-F	AGCACCTTCAAGAACACGG	114	NM_001163885.3
		FABP3-R	GACAAGTTTGCCTCCATCC	114	
100063687	*PKM*	PKM-F	GTTTGCGTCTTTCATCCG	120	NM_001143794.2
		PKM-R	AACCTCCGAACTCCCTCA	120	
100066121	*ALDOA*	ALDOA-F	AACCGACGCTTCTACCGC	144	XM_005598801.4
		ALDOA-R	GCCCTTGGATTTGATAACTT	144	
791234	*MYH7*	MYH7-F	AACGCCTTTGATGTGCTG	110	NM_001081758.1
		MYH7-R	TCTCGCTGCTTCTGCTTG	110	
791235	*MYH1*	MYH1-F	CTGGTCTCCTGGGGCTCCTA	72	NM_001081759.1
		MYH1-R	TGGCCTGGGTTCGGGTAA	72	
100060464	*EEF1A2*	18S-F	AGAAACGGCTACCACATCC	169	XM_023626927.2
		18S-R	CACCAGACTTGCCCTCCA	169	

**Table 2:** Real-Time Fluorescent Quantitative PCR experimental steps.

Reagent Name	Volume (μL)	Step	Time (Sec)	Cycles
2xqPCRmix	5.0	95 °C	30	
F primer (10 pmol/μL)	0.25	95 °C	10	40 cycles
R primer (10 pmol/μL)	0.25	60 °C	30	
DNA template	2.0	95 °C	15	
ddH_2_O	2.5	60 °C	60	Detect once every 0.5 °C increase in temperature
total	10.0	95 °C	15	

**Table 3:** Proportion of skeletal muscle fibers in different parts of Yili horses.

Part	Average Slow Muscle Area	Proportion of Slow Muscle Fiber Area
longissimus dorsi	1894.45 ± 385.76 ^Bb^	17.08 ± 3.98 ^Aa^
triceps brachii	1576.91 ± 673.28 ^ABab^	16.36 ± 7.35 ^Aa^
splenius muscle	2583.59 ± 449.98 ^Cc^	37.57 ± 4.69 ^Bb^
gluteus medius	1295.75 ± 284.77 ^Aa^	14.64 ± 5.49 ^Aa^

Note: Differences between columns indicated by different uppercase letters are highly significant (*p* < 0.01), while differences indicated by different lowercase letters are significant (*p* < 0.05).

**Table 4:** JJ vs. TZJ targeting differential metabolites.

Compounds	VIP	*p*-Value	Type
Glutamine	1.71	0.00	down
L-Asparagine	1.17	0.02	down
L-Alanine	1.42	0.00	down
L-citrulline	1.48	0.00	up
Ornithine	1.51	0.00	down
Arginine	1.48	0.00	down
L-Cystine	1.17	0.04	up
Lysine	1.62	0.00	down
Serine	1.60	0.00	down
L-Glutamate	1.54	0.00	down
Threonine	1.19	0.02	down
D-Glycerate	1.22	0.04	up
Gluconate	1.36	0.00	up
Glycolate	1.47	0.00	down
dCMP	1.31	0.02	up
Fumarate	1.52	0.00	down
L-Malic acid	1.08	0.04	down
L-Argininosuccinate	1.46	0.00	down
Pyruvic acid	1.26	0.03	up
Lactate	1.16	0.02	up
Alpha-Ketoglutaric Acid	1.08	0.04	down
Citric acid	1.31	0.02	down
Xylulose-5-phosphate	1.22	0.03	down
D-Ribose 5-phosphate	1.28	0.02	down
D-Ribulose 5-phosphate	1.28	0.02	down
D-Mannose 6-phosphate	1.07	0.03	up
D-Glucose 6-phosphate	1.26	0.01	up

## Data Availability

The original contributions presented in the study are included in the article, further inquiries can be directed to the corresponding author.

## Appendix A

The quality assessment of RNA sequencing data for 12 samples is shown in the table. As indicated, JJ group and TZJ group yielded 264,448,146 and 280,043,346 clean reads, respectively. Each sample contained over 5.98 G of clean bases, with R1 and R2 ends achieving Q20 and Q30 values exceeding 96.99% and 96.02%, respectively. The GC content of sample paired-end reads ranged from 48.79% to 51.30%. Q30 values at the R1 and R2 ends were above 96.99%:96.02% and 92.20%:90.50%, respectively. The GC content of the samples ranged from 48.79% to 51.34%. The transcriptome sequencing results are suitable for further research.

**Table A1:** Quality Assessment of mRNA Data.

Sample	Raw Data (bp)	Clean Data (bp)	AF_Q20 (%)	AF_Q30 (%)	AF_GC (%)
JJ1	13,199,480,700	13,122,366,300	98.06%	94.29%	53.45%
JJ2	12,318,236,100	12,236,089,545	98.10%	94.43%	52.73%
JJ4	12,538,311,900	12,445,278,760	98.19%	94.76%	54.85%
JJ6	12.358,093,500	12,257,883,689	97.89%	94.17%	55.86%
JJ7	13,921,296,300	13,798,455,360	97.87%	94.17%	56.48%
JJ9	12,238,812,600	12,155,905,199	98.01%	94.37%	55.42%
TZJ1	15,664,848,300	15,575,239,447	98.25%	94.81%	54.46%
TZJ2	13,394,916,600	13,291,996,474	98.17%	94.68%	54.99%
TZJ4	12,642,682,500	12,548,268,292	98.21%	94.86%	55.24%
TZJ6	12,549,490,800	12,464,847,291	98.09%	94.53%	55.09%
TZJ7	12,779,957,400	12,729,639,875	98.16%	94.71%	55.35%
TZJ9	13,679,158,200	13,562,642,434	97.89%	94.12%	55.63%

Mapping rate directly reflects transcriptome data utilization and the availability of reference genomes. The mapping rates of sequencing data from experimental samples against the reference genome are shown in the table. As indicated, the total reads and mapping rates for all 12 samples are comparable, confirming the suitability of the selected reference genome and normal data utilization.

**Table A2:** Comparison Results of Clean Reads and Reference Genome.

Sample	Total	Unmapped (%)	Unique Mapped (%)	Multiple Mapped (%)	Total Mapped (%)
JJ1	85,220,450	24,171,841 (28.36%)	53,329,465 (62.58%)	7,719,144 (9.06%)	61,048,609 (71.64%)
JJ2	79,839,962	16,434,587 (20.58%)	56,619,830 (70.92%)	6,785,545 (8.50%)	63,405,375 (79.42%)
JJ4	80,365,626	22,305,613 (27.76%)	47,878,326 (59.58%)	10,181,687 (12.67%)	58,060,013 (72.24%)
JJ6	79,024,234	20,471,147 (25.90%)	47,937,663 (60.66%)	10,615,424 (13.43%)	58,553,087 (74.10%)
JJ7	88,507,630	23,218,567 (26.23%)	53,935,327 (60.94%)	11,353,736 (12.83%)	65,289,063 (73.77%)
JJ9	78,170,250	27,655,376 (35.38%)	39,918,915 (51.07%)	10,595,959 (13.55%)	50,514,874 (64.62%)
TZJ1	100,602,282	26,253,104 (26.10%)	63,473,384 (63.09%)	10,875,794 (10.81%)	74,349,178 (73.90%)
TZJ2	85,659,920	24,533,391 (28.64%)	51,520,889 (60.15%)	9,605,640 (11.21%)	61,126,529 (71.36%)
TZJ4	81,896,358	26,561,246 (32.43%)	43,525,361 (53.15%)	11,809,751 (14.42%)	55,335,112 (67.57%)
TZJ6	81,062,762	21,788,362 (26.88%)	4,692,202 (57.88%)	12,352,371 (15.24%)	59,274,400 (73.12%)
TZJ7	82,591,530	29,121,524 (35.26%)	46,744,045 (56.60%)	6,725,961 (8.14%)	53,470,006 (64.74%)
TZJ9	86,962,266	30,874,280 (35.50%)	43,799,729 (50.37%)	12,288,257 (14.13%)	56087,986 (64.50%)

Sequencing saturation analysis is used to determine whether the sequencing depth of a sample has reached saturation. As the number of sequencing reads increases, the number of detected genes also rises. When the sequencing depth reaches a certain threshold, the growth rate of detected genes levels off, indicating that the number of detected genes is approaching saturation (Figure A1 is the JJ group, Figure A2 is the TZJ group).
